# Supplementary figures and images for: Identification of Potential Amblyomma americanum Antigens After Vaccination with Tick Extracellular Vesicles in White-Tailed Deer
Source: Vaccines (Basel). 2025 Mar 27;13(4):355. doi: 10.3390/vaccines13040355 (PMC12031118; doi:10.3390/vaccines13040355)

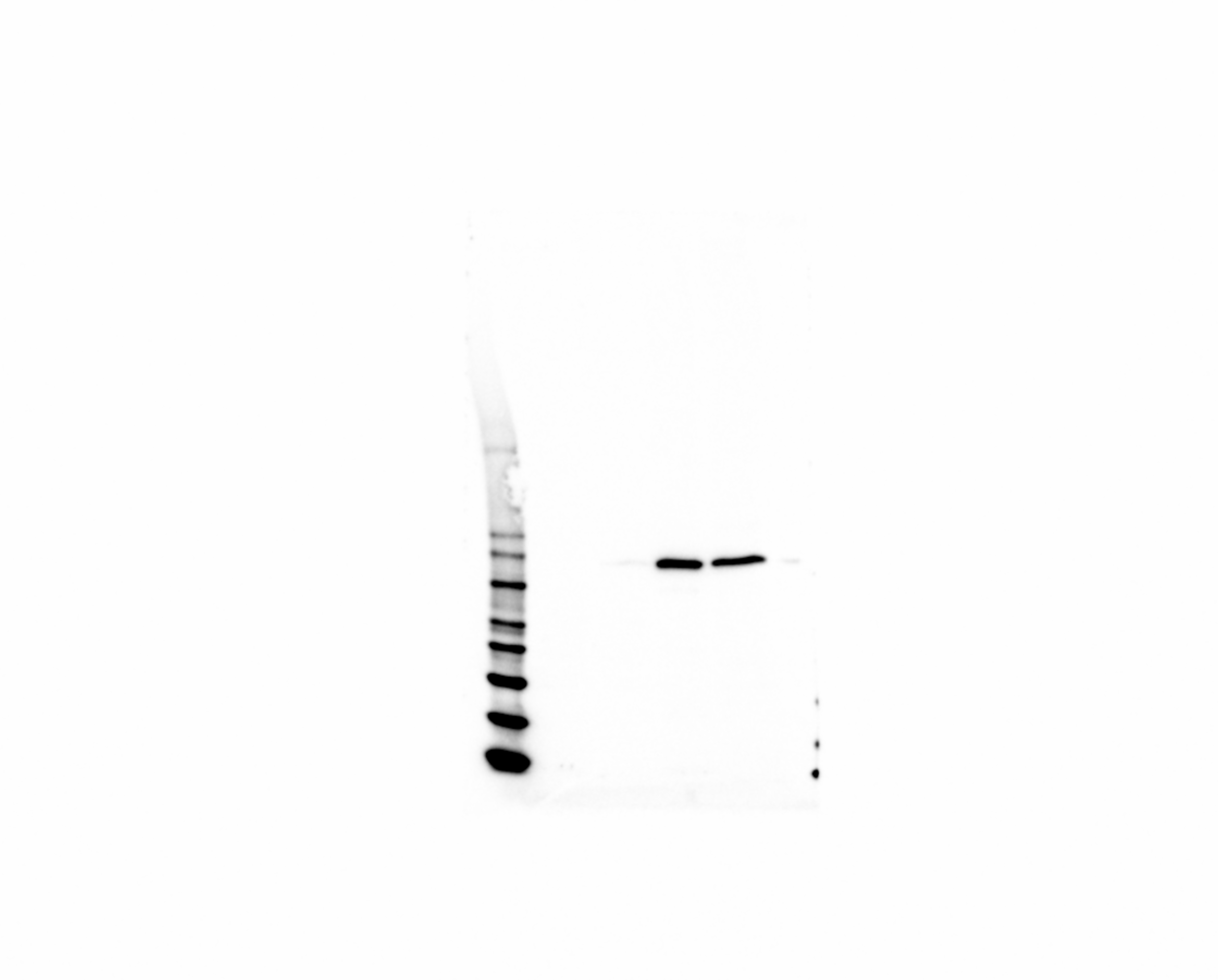

Supplement: Supplementary file 1 [file vaccines-13-00355-s001.zip › Calnexin_PUB_600.tif]

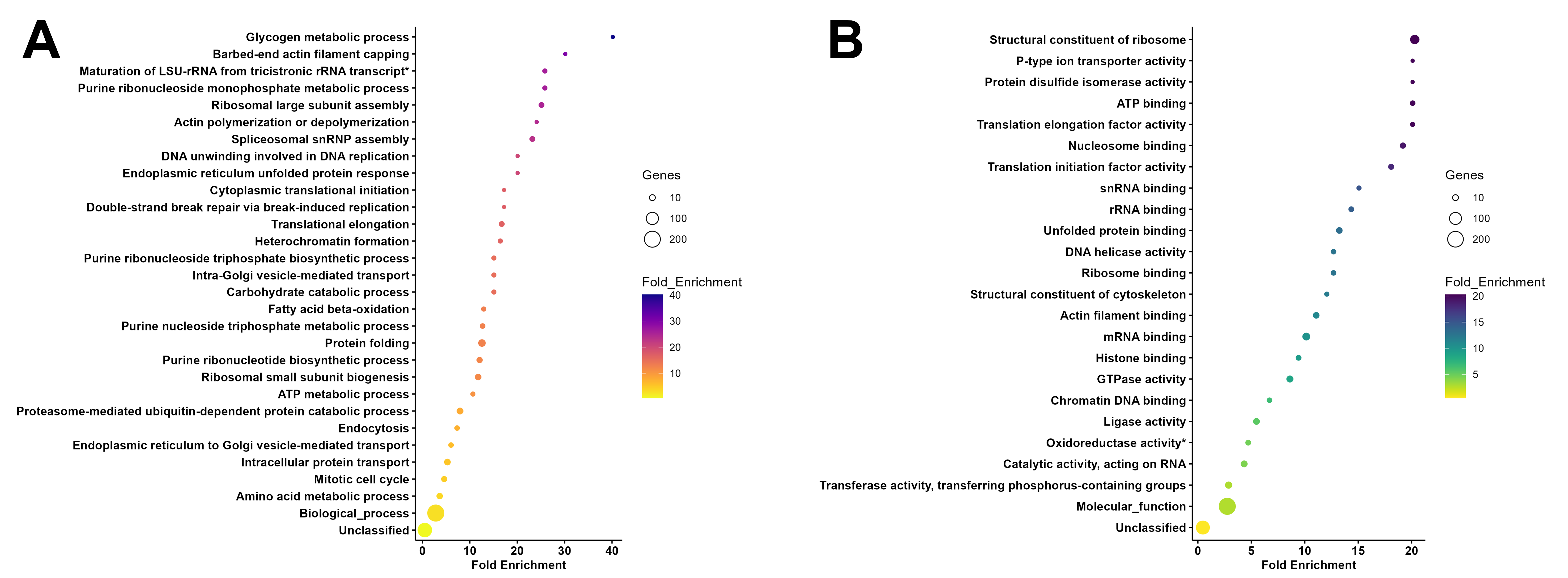

Supplement: Supplementary file 1 [file vaccines-13-00355-s001.zip › Figure S1. Enrichment analysis shared proteins MG SG EVs Amblyomma americanum.tif]

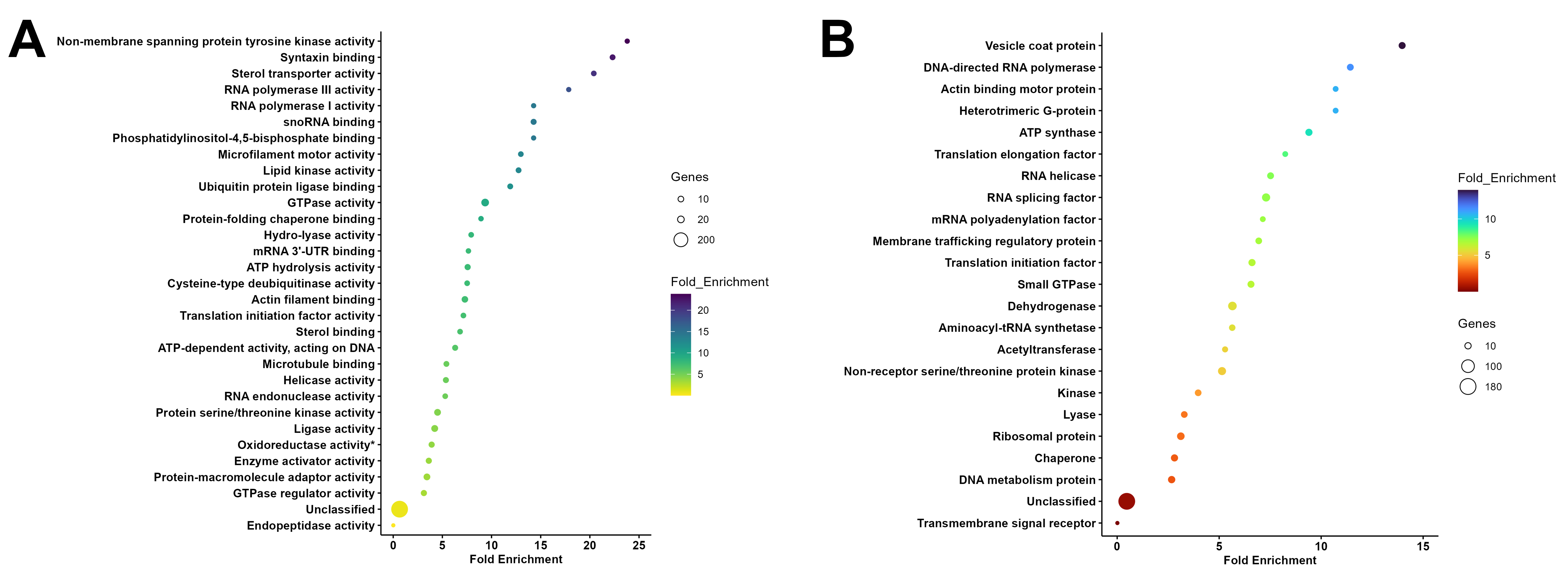

Supplement: Supplementary file 1 [file vaccines-13-00355-s001.zip › Figure S2. Enrichment analysis of unique proteins MG EVs Amblyomma americanum WTD.tif]

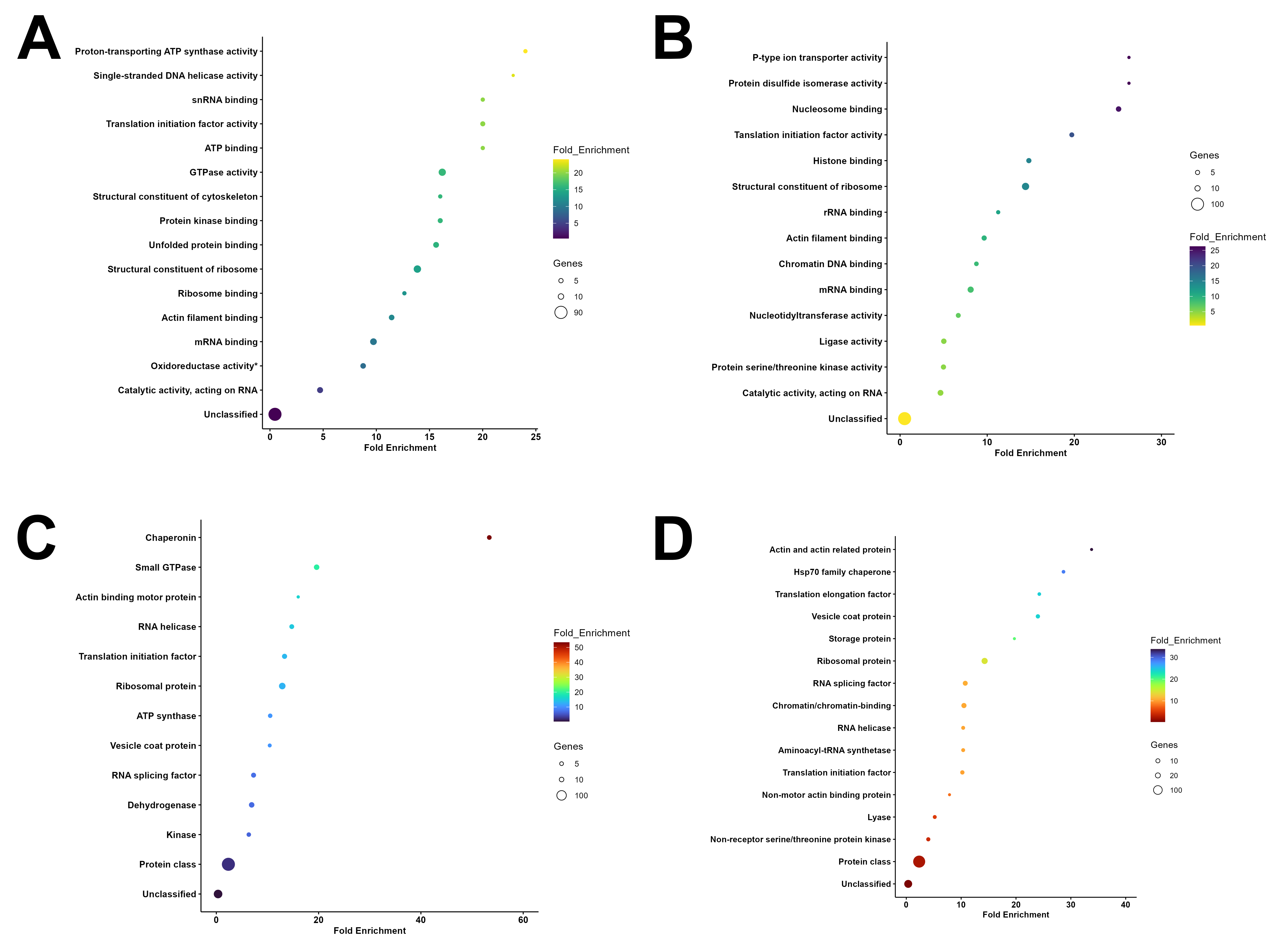

Supplement: Supplementary file 1 [file vaccines-13-00355-s001.zip › Figure S3. Enrichment analysis of differentially abundant proteins.tif]

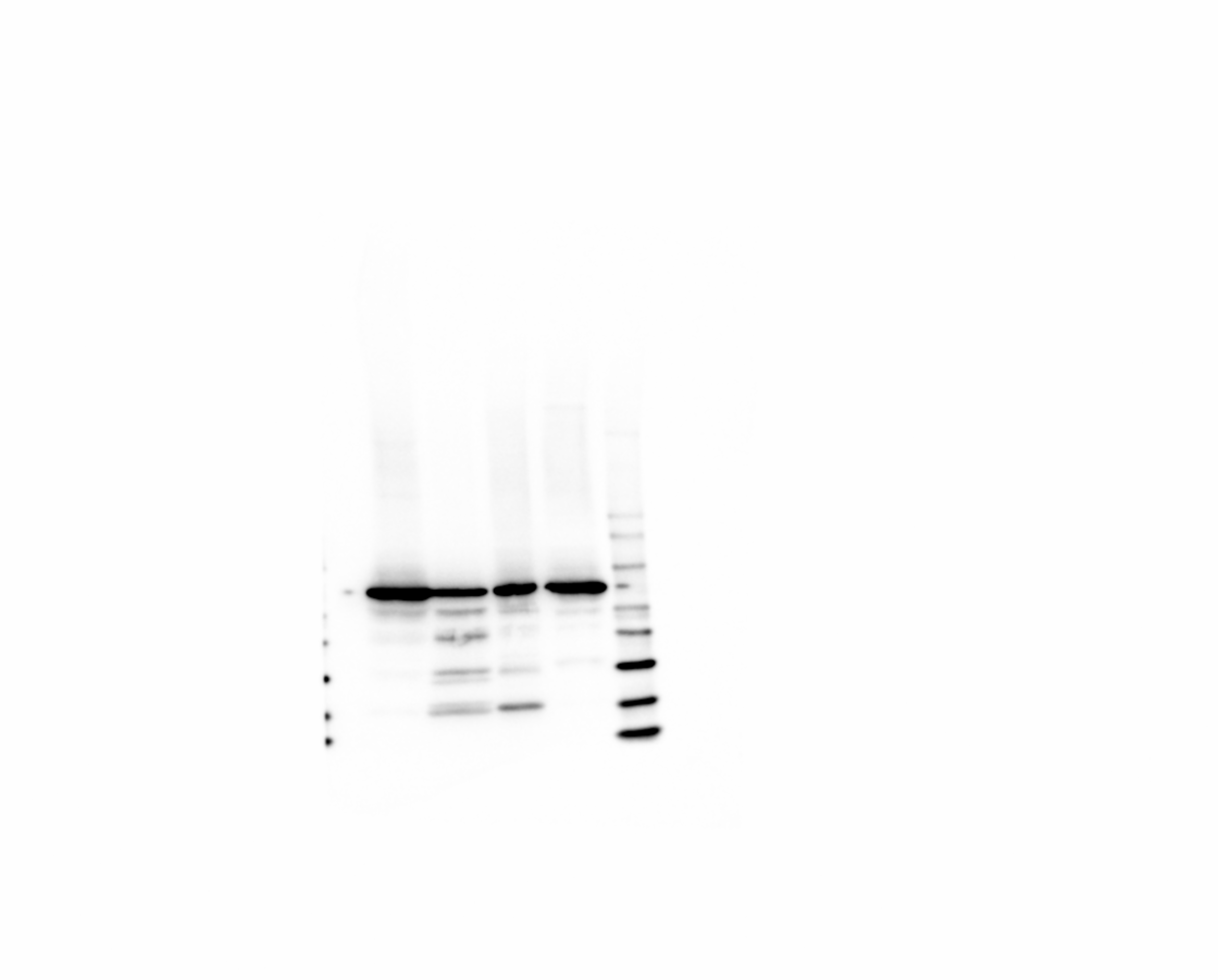

Supplement: Supplementary file 1 [file vaccines-13-00355-s001.zip › Hsp70_PUB_600.tif]
